# Targeting Renal Proximal Tubule Cells in Obesity-Related Glomerulopathy

**DOI:** 10.3390/ph16091256

**Published:** 2023-09-05

**Authors:** Muyao Ye, Ming Yang, Wenni Dai, Hao Li, Xun Zhou, Yinyin Chen, Liyu He

**Affiliations:** 1 Department of Nephrology, The Second Xiangya Hospital, Central South University, Hunan Key Laboratory of Kidney Disease and Blood Purification, Changsha 410011, China; yemuyao2064@csu.edu.cn (M.Y.); yangming940124@163.com (M.Y.);; 2 Department of Nephrology, Hunan Provincial People’s Hospital, The First Affiliated Hospital of Hunan Normal University, Changsha 410081, China; 3 Changsha Clinical Research, Changsha 410011, China

**Keywords:** renal proximal tubule cells, obesity-related glomerulopathy, lipid metabolism, oxidative stress

## Abstract

As a metabolic disorder, obesity can cause secondary kidney damage, which is called obesity-related glomerulopathy (ORG). As the incidence of obesity increases worldwide, so does the incidence of end-stage renal disease (ESRD) caused by ORGs. However, there is still a lack of effective strategies to prevent and delay the occurrence and development of ORG. Therefore, a deeper understanding and elaboration of the pathogenesis of ORG is conducive to the development of therapeutic drugs for ORG. Here, we review the characteristics of pathological lesions of ORG and describe the roles of lipid metabolism disorders and mitochondrial oxidative stress in the development of ORG. Finally, we summarize the current available drugs or compounds for the treatment of ORG and suggested that ameliorating renal lipid metabolism and mitochondrial function may be potential therapeutic targets for ORG.

## 1. Introduction

Kidney damage due to obesity, called obesity-related glomerulopathy (ORG), is now an important cause of chronic kidney disease and its incidence is increasing with the global obesity epidemic [1,2]. ORG has various histopathological alterations, including glomerular hypertrophy, focal segmental glomerulosclerosis, lipid deposition, etc. Positive oil red O lipid staining of renal biopsy tissue under a light microscope is considered the common manifestation of ORG. Recently, multiple studies showed that tubule injury is also involved in ORG [3,4]. Altered lipid metabolism leads to ectopic lipid accumulation, especially in the kidney, which plays a key role in ORG progression. Lipids can induce lipotoxicity in podocytes, renal proximal tubular cells (PTCs), and mesangial cells [5]. Renal lipotoxicity often leads to increased levels of oxidative stress, which in turn leads to renal cell apoptosis and fibrosis [6,7,8]. As a hypermetabolic site, renal PTCs are enriched with a large number of mitochondria to provide energy for their reabsorption of substances [9]. In the kidney of ORG, the oxidative stress level is increased due to the increased production of reactive oxygen species (ROS) and the suppressed FFA-induced antioxidant response in renal PTCs [10].

With continuous intensive research, the mechanism of ORG has experienced new advances, especially the important role of oxidative stress in proximal tubular injury. Since ORG is a metabolic disease, the lipid metabolism process in PTCs is similar to that of diabetic nephropathy (DN). There are more studies on renal tubular lesions in diabetes. With the gradual recognition of the importance of renal tubular injury in ORG, clinicians proposed ORN (obesity-related nephropathy) to highlight the injury of the tubulointerstitium, just like the conversion from DN to diabetic kidney disease (DKD). In this review, we cover different aspects of oxidative stress-related mechanisms in PTC and discuss the potential therapeutic target for ORG.

## 2. Histopathology of ORG

The histopathology of ORG contains glomerular, tubulointerstitial, and vascular lesions. Glomerular alterations of ORG are widely recognized as glomerular hypertrophy, focal segmental glomerulosclerosis, accompanied by segmental basement membrane thickening [11]. Some showed macrophage infiltration and upregulation of proinflammatory factors such as tumor necrosis factor α (TNF-α), monocyte chemotactic protein-1 (MCP-1), nuclear factor kappa-B (NF-κB), and profibrotic cytokines transforming growth factor-β (TGF-β) [5]. Lipid droplets were detected in both glomerular and PTCs, but predominantly in PTCs [12]. The hypertrophy and apoptosis of podocytes and mesangial cells caused glomerulomegaly [13]. Urinary alpha-1-acid glycoprotein (α1-AGP), albumin-to-creatinine ratio, and podocyte-specific proteins mRNA were tested as biomarkers of early glomerular damage in ORG [14].

Tubulointerstitial lesions contain tubular hypertrophy, atrophy, fibrosis, etc. Glomerular hypertrophy increases intra-glomerular pressure and drives glomerular filtration barrier injury, resulting in proteinuria. Glomerular hyperfiltration is also associated with tubular hypertrophy by the increased proximal tubular ultrafiltrate flow rate [15]. The majority of plasma-free fatty acid (FFA) is carried on albumin and provides ATP for the kidney by the mitochondrial β-oxidation of FFA. Albumin can be retrieved partly by the renal proximal tubules, resulting in intracellular lipid droplet deposition [16]. FFA-bound albumin-related oxidative stress is involved in the pathogenesis of PTC damage. Because the kidney is composed largely of tubular structures, tubular hypertrophy contributes to the relatively low glomerular density [17]. The vascular lesions are not specific in ORG, including dilation of the capillaries and glomerular arterioles around the vascular pole of glomeruli [11].

## 3. Mechanisms Involved in PTC Injury in the ORG

Lipoprotein and lipid metabolism abnormalities play a crucial role in the progres-sion of renal injury. The oxygen requirement of the kidney is very large, so the glomerular and PTC are very sensitive to hypoxia. In particular, renal PTC is highly dependent on aerobic metabolism [9]. Obese patients have endogenous hypoxia, which stimulates the secretion of inflammatory molecules [18]. The aggregation of inflammatory cells leads to increased local oxygen consumption, which in turn leads to the aggravation of hypoxia and injury. The disorder of lipid metabolism in ORG leads to excessive fat deposition in renal PTC and lipid metabolism ultimately also requires oxidative reaction in mitochondria to produce ATP, so the study of mitochondrial dysfunction is gradually being carried out. In addition, hypoxia caused by local mechanical compression caused by fat deposition has gradually attracted attention in ORG. This review will describe the hypoxia and oxidative stress injury of renal PTC in ORG from different aspects.

### 3.1. Renal Sinus Fat Compression Reduced Tubular Perfusion and Induced Renal Hypoxia

Fat deposition may occur in different areas of the kidney, including the perirenal area outside the renal capsule, the hilum, the renal sinus, and the retroperitoneal space [19]. Recently, many studies have focused on the renal sinus because of its special location. Fat deposition may occur in different areas of the kidney, including the perirenal area outside the renal capsule, the hilum, the renal sinus, and the retroperitoneal space. It locates in the center of the kidney, where fat is mostly deposited. Increased renal sinus fat (RSF) directly compresses various renal structures, resulting in tissue perfusion and tubular flow decrease [20]. The local ischemia and low tubular flow rate induce physical renal hypoxia. The serum kidney injury molecule-1 (sKIM-1) was elevated by the RSF accumulation, as a marker of renal tubular injury [21]. All the above reasons may stimulate increased renal sodium reabsorption. So, RSF increase was considered to be positively associated with increased renal hypertension and chronic kidney disease (CKD) development [22].

The increase in RSF is not specific in ORG; it also occurs in DN because of glucose intolerance [23]. The blood oxygen level-dependent magnetic resonance (MR) imaging showed that lipid accumulation in diabetic kidneys affected the renal cells’ oxygenation and made them more susceptible to renal hypoxia [24]. RSF increases in the early stage of diabetes and obesity and is associated with cardiovascular risk factors. This showed that RSF may be the link between metabolic disease and associated renal disease, even serving as an early non-invasive examination marker.

Weight loss can reduce fat deposition in the whole body, including the kidney. The renal cells oxygenate and make them more susceptible to renal hypoxia. Weight loss in obese and overweight people can reduce RSF [25] and further lower blood pressure and proteinuria. The plasma creatinine concentration and glomerular filtration rate (GFR) decreased significantly in the early stage of weight loss, but not significantly with long observation [26]. RSF can be a target of ORG prevention and monitoring.

### 3.2. Lipid Metabolism in PTC

**Figure 1:**
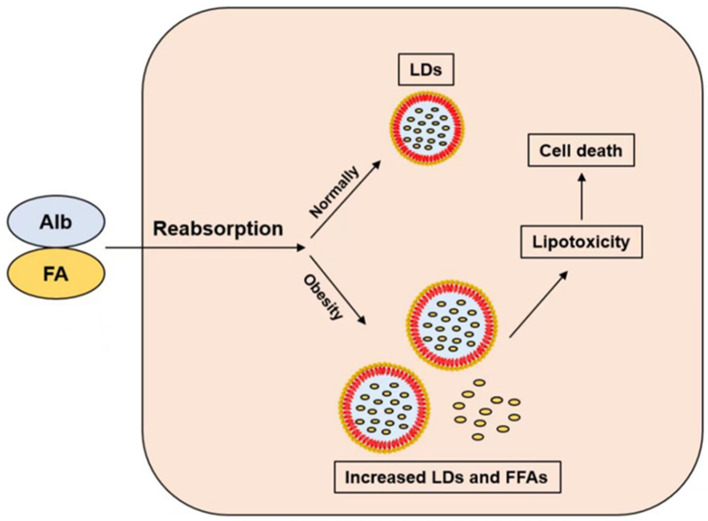
The albumin-bound FFA is reabsorbed by PTCs. FFAs are stored as LDs in normal persons, but increased LDs and FFAs can lead to lipotoxicity and cell death in obese patients. Abbreviation: FA: fatty acid; FFA: free fatty acid; Alb: albumin; LDs: lipid droplets.

FFA is the preferred energy source for PTC, which is more effective than glucose. Almost all plasma fatty acids are bound to albumin, with only a very small percentage being FFA. Under normal conditions, albumin cannot pass through the glomerular filtration barrier; a small amount of FFA is reabsorbed from ultrafiltrate by receptor-mediated endocytosis [27]. Almost all plasma fatty acids are bound to albumin, with only a very small percentage being FFA (Figure 1). After ingestion, FFA is converted to acyl-coenzyme A molecules and catalyzed by carnitine palmitoyl transferase-1 (CPT1) into mitochondria. According to the energy demand of cells, intracellular fatty acids are directed to mitochondrial β-oxidation, or stored as triglyceride in intracellular lipid droplets if excessive. In the process of FFA producing ATP, mitochondrial aerobic metabolism is crucial. Ischemia and hypoxia lead to decreased FFA oxidation and the conversion of FFA to triglyceride. The FFA in kidney is extracted from the circulation and requires more than half of the oxygen consumed by the kidneys for oxidation. The mitochondrial β-oxidation of FFA provides most of the ATP for the kidney [19]. The proximal tubules have high energy requirements and relatively small glycolytic capacity, which makes them very sensitive to hypoxia. Almost all plasma FFA is carried by albumin. Although weight loss is beneficial to obesity-related diseases in long-term observation, fasting may be detrimental to serum FFA levels and cause FFA-induced damage to renal PTC in a short period, especially on the premise of the damaged glomerular filtration barrier and proteinuria. The physiological role of insulin is the facilitation of glucose uptake and utilization, inhibition of FFA released by adipose tissue, and ultimately enhancement of lipogenesis. During fasting, the decreased blood glucose induced the decreased insulin level, thus promoting the decomposition of triacylglycerol in adipocytes into FFA. So, FFA is the energy source for various tissues. In both diabetic and non-diabetic subjects, fasting increases serum FFA levels [28]. In non-diabetic people, serum FFA levels rapidly decrease after meals. However, FFA remains high in diabetic and obese patients, who have impaired insulin action. Therefore, it is important to explore the mechanism of FFA-induced damage to renal PTCs and protect them from FFA-induced lipotoxicity.

FFAs are classified into monounsaturated, polyunsaturated, and saturated fatty acids. Saturated FFAs are highly cytotoxic among them. The increased saturated FFAs are considered to contribute to PTC damage and podocyte injury in diabetic nephropathy. Saturated FFAs damage PTCs via multiple mechanisms such as the TLR4-dependent pathway, DAG-PKCθ pathway, and oxidative stress, which act on NF-κB leading to inflammation and directly induce cell death or apoptosis [29]. In addition, the albumin-bound nonesterified fatty acids (NEFA, also known as FFA) that passed through the damaged glomerular filtration barrier can also be reabsorbed by the proximal tubule through fatty acid transporter-2 (FATP2), causing lipid accumulation and lipoapoptosis [29].

### 3.3. Lipotoxicity in the PTC

**Figure 2:**
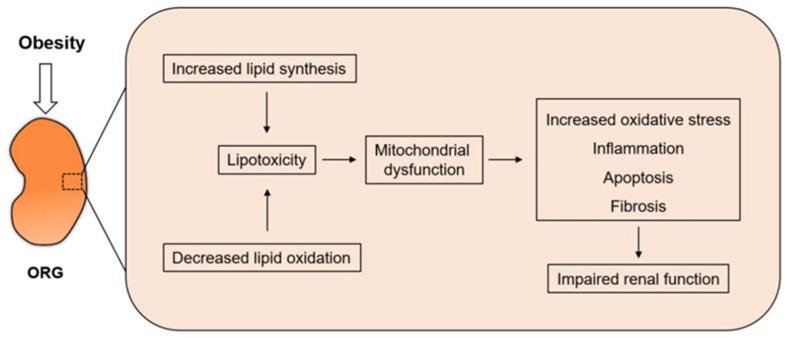
Increased lipid synthesis and decreased lipid oxidation occur simultaneously in obese patients. As mitochondria are the site of FFA undergoing β-oxidation to produce ATP, mitochondrial dysfunction induces increased oxidative stress and eventually leads to impaired renal function.

The intracellular accumulation of lipids in non-adipose tissue can cause lipotoxicity, resulting in cellular dysfunction and cell death. Most cells have limited carrying capacity for excessive intracellular lipids. Once the threshold and storage for lipid metabolism have been exceeded, the excessive lipid exerts lipotoxic effects. The released lipid metabolites can lead to malfunction of proteins, aberrant lipid modification, and membrane disruption. The ROS generation, endoplasmic reticulum (ER) stress, and transporters and signaling enzymes are activated; then, apoptosis is increased [30]. Renal lipotoxicity occurs in metabolism diseases, such as diabetic nephropathy and ORG. The lipids deposit mostly in the renal tubular epithelial cells, and toxic fatty acid metabolites induce inflammatory cytokine production, oxidative stress, ER stress, ROS generation, mitochondrial dysfunction, and autophagy in PTCs [31,32]. In a sense, the final result of most of the above mechanisms is to promote the oxidative stress of renal PTCs and eventually cause apoptosis (Figure 2). There are many studies on inflammatory cytokine in renal PTC of Fas-induced renal injury, such as interleukin 1β (IL-1β), IL-18, cluster of differentiation 36 (CD36), MCP-1, and NOD-like receptor thermal protein domain associated protein 3 (NLRP3) inflammasome [33,34]. With the massive recruitment of inflammatory cells, the oxygen needs to increase, leading to the production of ROS, which causes oxidative injury to proximal tubules. The activation of the NLRP3 inflammasome depends on oxidized low-density lipoprotein (ox-LDL)-induced generation of ROS depending on CD36 [35].

CD36 is also called scavenger receptor B2, and can mediate the uptake of protein-bound FAs by tissue cells in obesity and lipid metabolism [36]. In the kidney, CD36 mainly expresses in tubular epithelial, podocytes, and mesangial cells, where lipid deposition usually occurs, and lipid droplets can be detected by Oil Red O staining in kidney samples. Exposure to albumin and advanced oxidation protein products promotes the expression of CD36 in PTC. Moreover, CD36 may be regulated by peroxisome proliferator-activated receptor (PPAR)-γ, which belongs to fatty acid oxidation adjustment factors [37]. Dysregulation of PPAR activity affects the β-oxidation of FAs and is a potential cause of metabolic syndrome-related disorders, such as hyperlipidemia and insulin resistance. In addition, the advanced oxidation protein products (AOPPs) can induce lipotoxicity, apoptosis, and fibrosis via the CD36 receptor pathway in proximal tubular epithelial cells [38]. Carnitine palmitoyl transferase 2 (CPT2) can convert acylcarnitine to long-chain acyl-CoA and FAs and promote β-oxidation. ER stress inhibits CPT2 and in turn promotes lipid accumulation, leading to lipotoxicity [39]. The consequences of lipid deposition in the kidneys depend not only on the amount of lipids deposited, but also on the characteristics of the kinds of lipids that accumulate. Studies have shown significant increases in triglyceride and cholesterol levels in the kidneys of high-fat diet (HFD)-treated mice [40], as well as the accumulation of phospholipids [41]. Moreover, Lanzon et al. found significant differences in serum metabolomics in severely obese patients with and without CKD [42]. Most lipid types are increased in obese patients with kidney disease and serum short chain TG levels were negatively correlated with estimated glomerular filtration rate (eGFR) [42]. In addition, after weight-loss surgery, the renal function of CKD patients was significantly improved, and the levels of various lipids including unsaturated phosphatidylethanolamine were also significantly decreased [42].

Adenosine monophosphate-activated protein kinase (AMPK) is mostly expressed in cortical tubular epithelial cells and may lead to renal cell dysfunction and then develop into ORG. Since AMPK promotes fatty acid oxidation and inhibits fatty acid synthesis by phosphorylation of acetyl-CoA carboxylase, the decrease in AMPK inhibits the oxidation of FAs and induces renal lipid depositions in the proximal tubule in ORG. In addition, the markers of oxidative stress, the urinary hydrogen peroxide and 8-hydroxy-2′-deoxyguanosine (8-OHdG, a marker of oxidative damage) levels, increased in obesity [43]. HFD can activate renal AMPK, upregulate fatty acid oxidation (FAO) enzymes, and finally induce autophagy in the mouse PTC [44].

Macroautophagy/autophagy can protect the kidney from injury through lysosomal degradation. Some researchers considered that autophagy deficiency can lead to renal injury in obesity. Sustained high autophagic flux in PTC leads to metabolic syndrome, and lipid overload-stimulated macroautophagy/autophagy is a novel mechanism of lipotoxicity in PTC. Eicosapentaenoic acid (EPA) promoted lipid droplet formation and then migrated from the lipid droplet to the mitochondria for β-oxidation. EPA alleviated renal lipotoxicity via restored autophagic flux and the production of mitochondrial ROS [45,46]. Similarly, the abnormal autophagy mediated by Mas (the G-protein coupled receptor of angiotensin) is involved in the occurrence of lipid-induced tubular injury of obesity-related kidney diseases [47]. Other studies about oxidative stress injury in PTC are gradually being discovered. As mitochondria are β-oxidation sites, more studies focus on the oxidative stress of mitochondria.

### 3.4. Mitochondrial Dysfunction in Proximal Tubular Injury

Mitochondria are the site of FAs that undergo β-oxidation to produce ATP. Insufficient oxidation of intracellular fatty acids is a critical pathogenic factor causing renal lipotoxicity. Lipid overload in renal PTC causes mitochondrial dysfunction, ultimately causing the production of ROS and apoptosis. FFA-bound bovine serum albumin (BSA) stimulates ROS accumulation and expression of MCP-1 in PTC and can be ameliorated by overexpression of Sirtuin 3 (SIRT3), which is localized in mitochondria [48]. FAs-induced high levels of ROS lead to mitochondrial dysfunction in PTCs, which in turn promotes more ROS. Damaged mitochondria decrease fatty acid β-oxidation capacity and ATP synthesis, resulting in further lipid accumulation. Mitochondria-targeted antioxidant SS31 is specifically concentrated on the inner membrane of mitochondria, which is also the site for ROS production. SS31 reduced lipid accumulation and increased autophagy, restoring renal AMPK activity [5,49]. In addition, calcium overload in mitochondria affects the β-oxidation of FFA; the activation of the Mas receptor increased the protein expression of the voltage-dependent anion channel (VDAC1), which is located on the outer membrane of mitochondria facilitating calcium transport into mitochondria [47]. Animal studies showed that the loss of biliverdin reductase A (BVRA) in renal PTC caused impaired mitochondrial respiration and β-oxidation [50]. The intracellular lipid droplets are connected to mitochondria through a part of it, which is known as the peridroplet mitochondria [51,52]. The disorder of lipid metabolism affects the process of FA transport from lipid droplets to mitochondria, thus affecting the β-oxidation of FA and ATP production, or causing the overflow of FFA from lipid droplets, resulting in lipotoxicity [52]. However, the role of peridroplet mitochondria in renal PTC of ORG needs further research to be verified.

## 4. Treatment of ORG

### 4.1. Weight Loss

ORG is renal damage caused by obesity, so the preferred treatment is weight loss. The ways to lose weight include calorie restriction, physical exercise, and a good lifestyle, and when the effects of these methods are not sufficient, another way to lose weight is surgery [53,54]. Adequate physical exercise can stimulate muscle tissue to secrete large amounts of myokines [55,56,57], and our previous review also described in detail the protective effect of myokines in delaying the progression of kidney disease [58]. In addition to that, Rebelos et al. recruited 23 morbidly obese women and 15 age- and sex-matched non-obese controls to measure renal volume and radiodensity by computed tomography, and showed higher total renal blood flow and increased eGFR in the obese group than in the control; FFA uptake in the kidneys was about 50% higher in the obese group [59]. However, after bariatric surgery, the renal FFA uptake rate decreased, the renal hemodynamic changes were reversed, and the structural changes were improved in the obese group [59]. In addition, in a study conducted by Serra et al. to determine the long-term effects of substantial weight loss on renal function in morbidly obese patients with ORG lesions, it was found that 92 morbidly obese patients with mild obesity-related glomerulopathy who had substantial weight loss after bariatric surgery had normal renal function confirmed by renal biopsy [60]. In addition, blood pressure decreased early after surgery and remained stable thereafter; creatinine clearance decreased during the first 2 years, increased slightly after 5 years, and then remained stable. Moreover, the decreases in serum creatinine and proteinuria levels occurred throughout the follow-up period [60]. Although bariatric surgery may preserve renal function through weight loss, the procedure carries certain risks and complications that warrant further surgical evaluation.

### 4.2. RAAS Inhibitors

There is increasing evidence that abnormal glomerular filtration is one of the pathogeneses of ORG. In the obese state, the number of functional nephrons is reduced, and compensatory changes in the kidney lead to vasodilatation of afferent arterioles and increased intraglomerular hydrostatic pressure, which eventually lead to hyperfiltration [61,62]. Given the central role of RAAS in the pathogenesis of ORG, another effective therapeutic approach is to block the activation of the RAAS system using angiotensin-converting enzyme inhibitors (ACEIs) or angiotensin II type I receptor blockers (ARBs). In a post hoc analysis assessing the efficacy of ramipril in a renal disease (REIN) trial, a greater reduction in the risk of renal events (ESRD or doubling of serum creatinine) with ramipril was found in obese patients compared with non-obese patients [63]. Moreover, ramipril can also increase insulin sensitivity in non-diabetic obese patients, which is a key factor in promoting ORG [64]. Although studies specifically targeting non-diabetic obese subjects are limited, the protective effect of RASS inhibitors in ORG is expected as it is an important treatment for reducing urinary protein in kidney disease to delay its progression.

### 4.3. Maintenance of Lipid Metabolic Homeostasis

Lipid metabolism disorder is also an important risk factor for ORG. Therefore, lipid-lowering is a therapeutic modality to relieve ORG. Chen et al. have demonstrated more severe lipid accumulation and inflammation and aggravated kidney injury in angiotensin-converting enzyme 2 (ACE2) knockout ORG mouse model, while ACE2 overexpression activated the NF-E2-related factor 2 (Nrf2) pathway and alleviated kidney pathological injury by reducing lipid deposition and inflammatory response [65]. Statins are a class of lipid-lowering drugs commonly used in the clinic. Studies have shown that simvastatin intervention effectively reduces the levels of tissue factor (TF) and plasminogen activator inhibitor-1 (PAI-1) in mesangial cells [66]. This means that simvastatin plays a kidney-protective role by inhibiting the formation of microthrombus in hypercholesterolemic conditions. Similarly, proprotein convertase subtilisin/kexin type 9 inhibitors (PCSK9i) are also a class of effective lipid-lowering drugs and side effects are less common in patients with CKD [67]. In the future, it may also serve as a therapeutic target for maintaining lipid homeostasis in ORG patients. Resveratrol is an anticancer hormone mainly found in mulberry, peanut, grape, and other plants. It has a variety of biological activities such as antioxidant, anti-inflammatory, cardiovascular protection, and anti-aging [68]. It was reported that resveratrol could reduce renal lipid deposition by regulating the junctional adhesion molecule-like (JAML)/sirtuin 1 (Sirt1) lipid synthesis pathway, thereby reducing renal injury in diabetic nephropathy state [69]. What is more, resveratrol also increased the expression of adiponectin receptor 1 (AdipoR1) and AdipoR2 in renal PTC [70]. Han et al. reported that when renal AdipoR1 is activated, it can activate the AMPK/lipophagy signaling pathway in cells, thereby alleviating lipid deposition in DN [71]. Moreover, pterostilbene, a methylated derivative of pterostilbene, also showed lipid-regulating effects. Gu et al. have shown that pterostilbene could inhibit the expression of sterol regulatory element binding transcription factor 1 (SREBP-1) and FAS protein and downregulate TGF-β1 and p-smad3 expression, and finally relieve ectopic lipid deposition, and reduce renal tubular injury and fibrosis in the kidney of high-fat-diet-fed mice [72]. In addition to these compounds, it has also been shown that mitochondria-associated ER membranes (MAMs) are indispensable for the maintenance of cellular lipid metabolism. MAMs are the subcellular structure that mediates the exchange of signals and substances between mitochondria and ER [73,74]. One of its major functions is to participate in intracellular lipid metabolism and synthesis [75]. Abnormality of its structure and function can lead to a disorder of intracellular lipid metabolism. In terms of kidney disease, the degree of its structural destruction is also closely related to renal lipid deposition in patients with DN [76]. However, there is no study on the structure and function of MAMs in ORG, but MAMs may be a potential research direction for lipid kidney injury in ORG in the future due to their importance in lipid metabolism.

### 4.4. Sodium-Glucose Cotransporter 2 Inhibitors (SGLT2i)

SGLT2i is a new class of oral hypoglycemic drug that has received great attention in recent years; it can inhibit the reabsorption of glucose by the kidney and promote the excretion of glucose in urine [77,78]. Several SGLT2 inhibitors, including empagliflozin, dapagliflozin, and canagliflozin, have been approved for clinical use [79,80,81]. Several studies have confirmed that SGLT2i plays a protective role in delaying the progression of kidney diseases [82,83,84]. In addition to its direct reno-protective effects, SGLT2i has also been shown to significantly reduce body weight, potentially in part by increasing energy expenditure and enhancing fatty acid oxidation [85]. Moreover, it may also alleviate ORG kidney injury by reducing renal lipid deposition, inflammation, and oxidative stress [86].

### 4.5. Adipokines

Excessive and unhealthy obesity is the main onset factor of ORG. Under these conditions, there is an unhealthy expansion of adipose tissue, and both structure and function are affected to some extent. In the human body, adipose tissue not only serves as a storage site for fat but also secretes a series of proteins called adipokines that affect distal tissues or organs through endocrine action [87,88]. Leptin was discovered in 1994 and is mainly released by white adipose tissue [89]. After it is synthesized and secreted by adipose tissue, it circulates to the hypothalamus through the blood and binds to specific receptors, then promotes the anorexigenic neuropeptide circuits and inhibits orexigenic neuropeptide circuits and leads to a decrease in food intake and an increase in energy expenditure [90,91,92]. Leptin knockout mice are commonly used as an obesity mouse model (ob/ob mice) [93,94]. In addition to leptin, adiponectin is also an adipokine secreted mainly by adipose tissue [95,96]. Xu et al. found decreased adiponectin expression in serum samples from HFD-fed mice, accompanied by downregulation of podocyte markers and increased levels of inflammation and apoptosis [97]. The intervention of adiponectin significantly alleviated renal injury, while inhibition of adiponectin expression aggravated renal inflammation, oxidative stress, and apoptosis [97]. To date, more than one hundred adipokines have been characterized, and their functions in kidney disease have been described in detail in our previous review [98]. Although the current research on adipokines is still in its infancy, the deepening of research on their role in the progression of kidney disease, especially ORG, is worth looking forward to.

### 4.6. Mitochondrial Homeostasis

Given the importance of mitochondrial disorders in ORG, maintaining the function of mitochondria is also a potential treatment for ORG therapy. Intervention with 1,2-Distearoyl-sn-glycero-3-phosphoethanolamine-(polyethylene glycol)-ss31 (SS-31) (a mitochondrial protective peptide) improved the mitochondrial structure of renal cells, upregulated AMPK activity, and reduced intracellular lipid accumulation, endoplasmic reticulum stress, and apoptosis in the kidney of mice fed an HFD [5]. A similar result was also observed that INT-777 could induce mitochondrial biogenesis, reduce oxidative stress levels, and increase fatty acid β-oxidation to relieve obesity-related kidney injury [99]. In the future, more specific mitochondrial protective agents need to be sought to further elucidate the importance of mitochondria in the development of ORG.

### 4.7. Others

In addition to the treatments mentioned above, some drugs have also been reported to prevent or delay the progression of ORG. Glucagon-like peptide-1 receptor agonists (GLP-1 RAs) could also slow the progress of ORG in different ways. On the one hand, GLP-1 RAs can promote urinary sodium excretion by inhibiting Na/H exchange 3 in the proximal renal tubules, and thus inhibit the activation of RAAS [100]. On the other hand, it can also effectively reduce the weight of obese patients, whether they have diabetes or not [101,102]. These characteristics suggest its feasibility in the treatment of ORG. Sulforaphane is a bioactive ingredient found in abundance in cauliflower, kale, and other cruciferous plants. It could ameliorate insulin resistance and glucose intolerance in HFD-induced obese mice [103]. Moreover, it also could inhibit malondialdehyde protein (MDA) accumulation induced by obesity and increase superoxide dismutase (SOD) levels, thus reducing obesity-related injury [104]. A recent study also showed that sulforaphane intervention reduced body weight, organ-associated fat weight, and urinary albumin/creatinine ratio in obese mice [105]. Furthermore, sulforaphane also upregulated the specific protein expression level of the podocyte and improved the podocyte function [105]. Moreover, other compounds, such as eicosapentaenoic acid [45], Coptidis Rhizoma [106], and lipoxin A4 [107] may also be potential targets for ORG therapy (Table 1).

## 5. Conclusions and Perspectives

As the number of obese people worldwide increases, the prevalence of ORG will gradually increase. Many factors are involved in the occurrence and development of ORG, while lipid metabolism disorder plays a major role in this process. In this review, we summarize the pathogenesis of ORG and current treatment options. Although a comprehensive understanding of the pathogenesis of ORG has been achieved, there are still many problems that need to be solved in the future for more effective prevention and treatment of ORG. At present, there is still a lack of early diagnostic markers for ORG, which makes the early diagnosis of ORG difficult. In addition, the effectiveness and feasibility of bariatric surgery in ORG remain to be validated. Once these outstanding issues are resolved, ORG can be effectively prevented and treated.

## Figures and Tables

**Table 1:** Potential treatments or drugs for obesity-related glomerulopathy.

Measures/Compounds	Categories	Ref.
Weight loss	Exercise	[54]
	Bariatric surgery	[60]
RAAS inhibitors	Ramipril	[63]
Lipid regulation	Simvastatin	[66]
	PCSK9i	[67]
	Resveratrol	[69]
	Pterostilbene	[72]
SGLT2i	Dapagliflozin	[86]
	Empagliflozin	[86]
Others	SS-31	[5]
	INT-777	[99]
	Sulforaphane	[105]
	Eicosapentaenoic acid	[45]
	Coptidis Rhizoma	[106]
	Lipoxin A4	[107]

## Data Availability

Data sharing is not applicable.

## References

[1] Kambham N., Markowitz G.S., Valeri A.M., Lin J., D’Agati V.D. (2001). Obesity-related glomerulopathy: An emerging epidemic. Kidney Int..

[2] Salvatore S.P., Chevalier J.M., Kuo S.F., Audia P.F., Seshan S.V. (2017). Kidney disease in patients with obesity: It is not always obesity-related glomerulopathy alone. Obes. Res. Clin. Pract..

[3] Ostalska-Nowicka D., Mackowiak-Lewandowicz K., Perek B., Zaorska K., Zachwieja J., Nowicki M. (2019). Megalin—A facultative marker of obesity-related glomerulopathy in children. J. Biol. Regul. Homeost. Agents.

[4] Sandino J., Martín-Taboada M., Medina-Gómez G., Vila-Bedmar R., Morales E. (2022). Novel Insights in the Physiopathology and Management of Obesity-Related Kidney Disease. Nutrients.

[5] Szeto H.H., Liu S., Soong Y., Alam N., Prusky G.T., Seshan S.V. (2016). Protection of mitochondria prevents high-fat diet-induced glomerulopathy and proximal tubular injury. Kidney Int..

[6] Escasany E., Izquierdo-Lahuerta A., Medina-Gomez G. (2019). Underlying Mechanisms of Renal Lipotoxicity in Obesity. Nephron.

[7] Martin-Taboada M., Vila-Bedmar R., Medina-Gómez G. (2021). From Obesity to Chronic Kidney Disease: How Can Adipose Tissue Affect Renal Function?. Nephron.

[8] Chen D., Ruan X., Liu Y., He Y. (2022). HMGCS2 silencing attenuates high glucose-induced in vitro diabetic cardiomyopathy by increasing cell viability, and inhibiting apoptosis, inflammation, and oxidative stress. Bioengineered.

[9] Gilbert R.E. (2017). Proximal Tubulopathy: Prime Mover and Key Therapeutic Target in Diabetic Kidney Disease. Diabetes.

[10] Arany I., Hall S., Reed D.K., Dixit M. (2016). The pro-oxidant gene p66shc increases nicotine exposure-induced lipotoxic oxidative stress in renal proximal tubule cells. Mol. Med. Rep..

[11] Tsuboi N., Okabayashi Y. (2021). The Renal Pathology of Obesity: Structure-Function Correlations. Semin. Nephrol..

[12] Bobulescu I.A., Lotan Y., Zhang J., Rosenthal T.R., Rogers J.T., Adams-Huet B., Sakhaee K., Moe O.W. (2014). Triglycerides in the human kidney cortex: Relationship with body size. PLoS ONE.

[13] D’Agati V.D., Chagnac A., De Vries A.P., Levi M., Porrini E., Herman-Edelstein M., Praga M. (2016). Obesity-related glomerulopathy: Clinical and pathologic characteristics and pathogenesis. Nat. Rev. Nephrol..

[14] Medyńska A., Chrzanowska J., Kościelska-Kasprzak K., Bartoszek D., Żabińska M., Zwolińska D. (2021). Alpha-1 Acid Glycoprotein and Podocin Mrna as Novel Biomarkers for Early Glomerular Injury in Obese Children. J. Clin. Med..

[15] Chagnac A., Zingerman B., Rozen-Zvi B., Herman-Edelstein M. (2019). Consequences of Glomerular Hyperfiltration: The Role of Physical Forces in the Pathogenesis of Chronic Kidney Disease in Diabetes and Obesity. Nephron.

[16] Bobulescu I.A. (2010). Renal lipid metabolism and lipotoxicity. Curr. Opin. Nephrol. Hypertens..

[17] Okabayashi Y., Tsuboi N., Sasaki T., Haruhara K., Kanzaki G., Koike K., Shimizu A., D’Agati V.D., Yokoo T. (2020). Single-Nephron GFR in Patients with Obesity-Related Glomerulopathy. Kidney Int. Rep..

[18] Wang R., Sun Q., Wu X., Zhang Y., Xing X., Lin K., Feng Y., Wang M., Wang Y., Wang R. (2022). Hypoxia as a Double-Edged Sword to Combat Obesity and Comorbidities. Cells.

[19] Mende C., Einhorn D. (2022). Fatty kidney disease: The importance of ectopic fat deposition and the potential value of imaging. J. Diabetes.

[20] Lamacchia O., Nicastro V., Camarchio D., Valente U., Grisorio R., Gesualdo L., Cignarelli M. (2011). Para- and perirenal fat thickness is an independent predictor of chronic kidney disease, increased renal resistance index and hyperuricaemia in type-2 diabetic patients. Nephrol. Dial. Transplant..

[21] Krievina G., Tretjakovs P., Skuja I., Silina V., Keisa L., Krievina D., Bahs G. (2016). Ectopic Adipose Tissue Storage in the Left and the Right Renal Sinus Is Asymmetric and Associated with Serum Kidney Injury Molecule-1 and Fibroblast Growth Factor-21 Levels Increase. Ebiomedicine.

[22] Spit K.A., Muskiet M.H.A., Tonneijck L., Smits M.M., Kramer M.H.H., Joles J.A., De Boer A., Van Raalte D.H. (2020). Renal sinus fat and renal hemodynamics: A cross-sectional analysis. Magma.

[23] Notohamiprodjo M., Goepfert M., Will S., Lorbeer R., Schick F., Rathmann W., Martirosian P., Peters A., Müller-Peltzer K., Helck A. (2020). Renal and renal sinus fat volumes as quantified by magnetic resonance imaging in subjects with prediabetes, diabetes, and normal glucose tolerance. PLoS ONE.

[24] Peng X.G., Bai Y.Y., Fang F., Wang X.Y., Mao H., Teng G.J., Ju S. (2013). Renal lipids and oxygenation in diabetic mice: Noninvasive quantification with MR imaging. Radiology.

[25] Zelicha H., Schwarzfuchs D., Shelef I., Gepner Y., Tsaban G., Tene L., Yaskolka Meir A., Bilitzky A., Komy O., Cohen N. (2018). Changes of renal sinus fat and renal parenchymal fat during an 18-month randomized weight loss trial. Clin. Nutr..

[26] Spurny M., Jiang Y., Sowah S.A., Nonnenmacher T., Schübel R., Kirsten R., Johnson T., Von Stackelberg O., Ulrich C.M., Kaaks R. (2022). Changes in Kidney Fat upon Dietary-Induced Weight Loss. Nutrients.

[27] Long K.R., Rbaibi Y., Gliozzi M.L., Ren Q., Weisz O.A. (2020). Differential kidney proximal tubule cell responses to protein overload by albumin and its ligands. Am. J. Physiol. Renal Physiol..

[28] Kume S., Maegawa H. (2020). Lipotoxicity, Nutrient-Sensing Signals, and Autophagy in Diabetic Nephropathy. JMA J..

[29] Khan S., Cabral P.D., Schilling W.P., Schmidt Z.W., Uddin A.N., Gingras A., Madhavan S.M., Garvin J.L., Schelling J.R. (2018). Kidney Proximal Tubule Lipoapoptosis Is Regulated by Fatty Acid Transporter-2 (FATP2). J. Am. Soc. Nephrol..

[30] Schelling J.R. (2022). The Contribution of Lipotoxicity to Diabetic Kidney Disease. Cells.

[31] Tanaka Y., Kume S., Araki H., Nakazawa J., Chin-Kanasaki M., Araki S., Nakagawa F., Koya D., Haneda M., Maegawa H. (2015). 1-Methylnicotinamide ameliorates lipotoxicity-induced oxidative stress and cell death in kidney proximal tubular cells. Free Radic. Biol. Med..

[32] Reverte V., Gogulamudi V.R., Rosales C.B., Musial D.C., Gonsalez S.R., Parra-Vitela A.J., Galeas-Pena M., Sure V.N., Visniauskas B., Lindsey S.H. (2020). Urinary angiotensinogen increases in the absence of overt renal injury in high fat diet-induced type 2 diabetic mice. J. Diabetes Complicat..

[33] Li L.C., Yang J.L., Lee W.C., Chen J.B., Lee C.T., Wang P.W., Vaghese Z., Chen W.Y. (2018). Palmitate aggravates proteinuria-induced cell death and inflammation via CD36-inflammasome axis in the proximal tubular cells of obese mice. Am. J. Physiol. Renal Physiol..

[34] Tanaka Y., Kume S., Chin-Kanasaki M., Araki H., Araki S.I., Ugi S., Sugaya T., Uzu T., Maegawa H. (2016). Renoprotective effect of DPP-4 inhibitors against free fatty acid-bound albumin-induced renal proximal tubular cell injury. Biochem. Biophys. Res. Commun..

[35] Liu W., Yin Y., Zhou Z., He M., Dai Y. (2014). Oxldl-Induced IL-1 Beta secretion promoting foam cells formation was mainly via CD36 mediated ROS production leading to NLRP3 inflammasome activation. Inflamm. Res..

[36] Wang Y., Zhou X.O., Zhang Y., Gao P.J., Zhu D.L. (2012). Association of the CD36 gene with impaired glucose tolerance, impaired fasting glucose, type-2 diabetes, and lipid metabolism in essential hypertensive patients. Genet. Mol. Res..

[37] Huang C.C., Chou C.A., Chen W.Y., Yang J.L., Lee W.C., Chen J.B., Lee C.T., Li L.C. (2021). Empagliflozin Ameliorates Free Fatty Acid Induced-Lipotoxicity in Renal Proximal Tubular Cells via the Pparγ/CD36 Pathway in Obese Mice. Int. J. Mol. Sci..

[38] Li X., Zhang T., Geng J., Wu Z., Xu L., Liu J., Tian J., Zhou Z., Nie J., Bai X. (2019). Advanced Oxidation Protein Products Promote Lipotoxicity and Tubulointerstitial Fibrosis via CD36/β-Catenin Pathway in Diabetic Nephropathy. Antioxid. Redox Signal.

[39] Rinaldi A., Lazareth H., Poindessous V., Nemazanyy I., Sampaio J.L., Malpetti D., Bignon Y., Naesens M., Rabant M., Anglicheau D. (2022). Impaired fatty acid metabolism perpetuates lipotoxicity along the transition to chronic kidney injury. JCI Insight.

[40] Sun Y., Ge X., Li X., He J., Wei X., Du J., Sun J., Li X., Xun Z., Liu W. (2020). High-fat diet promotes renal injury by inducing oxidative stress and mitochondrial dysfunction. Cell Death Dis..

[41] Yamamoto T., Takabatake Y., Takahashi A., Kimura T., Namba T., Matsuda J., Minami S., Kaimori J.Y., Matsui I., Matsusaka T. (2017). High-Fat Diet-Induced Lysosomal Dysfunction and Impaired Autophagic Flux Contribute to Lipotoxicity in the Kidney. J. Am. Soc. Nephrol..

[42] Lanzon B., Martin-Taboada M., Castro-Alves V., Vila-Bedmar R., González De Pablos I., Duberg D., Gomez P., Rodriguez E., Orešič M., Hyötyläinen T. (2021). Lipidomic and Metabolomic Signature of Progression of Chronic Kidney Disease in Patients with Severe Obesity. Metabolites.

[43] Juszczak F., Vlassembrouck M., Botton O., Zwakhals T., Decarnoncle M., Tassin A., Caron N., Declèves A.E. (2020). Delayed Exercise Training Improves Obesity-Induced Chronic Kidney Disease by Activating AMPK Pathway in High-Fat Diet-Fed Mice. Int. J. Mol. Sci..

[44] Sohn M., Kim K., Uddin M.J., Lee G., Hwang I., Kang H., Kim H., Lee J.H., Ha H. (2017). Delayed treatment with fenofibrate protects against high-fat diet-induced kidney injury in mice: The possible role of AMPK autophagy. Am. J. Physiol. Renal Physiol..

[45] Yamamoto T., Takabatake Y., Minami S., Sakai S., Fujimura R., Takahashi A., Namba-Hamano T., Matsuda J., Kimura T., Matsui I. (2021). Eicosapentaenoic acid attenuates renal lipotoxicity by restoring autophagic flux. Autophagy.

[46] Matsuda J., Takahashi A., Takabatake Y., Sakai S., Minami S., Yamamoto T., Fujimura R., Namba-Hamano T., Yonishi H., Nakamura J. (2020). Metabolic effects of RUBCN/rubicon deficiency in kidney proximal tubular epithelial cells. Autophagy.

[47] Kong Y., Zhao X., Qiu M., Lin Y., Feng P., Li S., Liang B., Zhu Q., Huang H., Li C. (2021). Tubular mas receptor mediates lipid-induced kidney injury. Cell Death Dis..

[48] Koyama T., Kume S., Koya D., Araki S., Isshiki K., Chin-Kanasaki M., Sugimoto T., Haneda M., Sugaya T., Kashiwagi A. (2011). SIRT3 attenuates palmitate-induced ROS production and inflammation in proximal tubular cells. Free Radic. Biol. Med..

[49] Tang C., Cai J., Dong Z. (2016). Mitochondrial dysfunction in obesity-related kidney disease: A novel therapeutic target. Kidney Int..

[50] Adeosun S.O., Gordon D.M., Weeks M.F., Moore K.H., Hall J.E., Hinds T.D., Stec D.E. (2018). Loss of biliverdin reductase—A promotes lipid accumulation and lipotoxicity in mouse proximal tubule cells. Am. J. Physiol. Renal Physiol..

[51] Acín-Perez R., Petcherski A., Veliova M., Benador I.Y., Assali E.A., Colleluori G., Cinti S., Brownstein A.J., Baghdasarian S., Livhits M.J. (2021). Recruitment and remodeling of peridroplet mitochondria in human adipose tissue. Redox Biol..

[52] Yang M., Luo S., Yang J., Chen W., He L., Liu D., Zhao L., Wang X. (2022). Lipid droplet—mitochondria coupling: A novel lipid metabolism regulatory hub in diabetic nephropathy. Front. Endocrinol..

[53] Benaiges D., Goday A., Pedro-Botet J., Más A., Chillarón J.J., Flores-Le Roux J.A. (2015). Bariatric surgery: To whom and when?. Minerva Endocrinol..

[54] O’Brien P.E., Hindle A., Brennan L., Skinner S., Burton P., Smith A., Crosthwaite G., Brown W. (2019). Long-term outcomes after bariatric surgery: A systematic review and meta-analysis of weight loss at 10 or more years for all bariatric procedures and a single-centre review of 20-year outcomes after adjustable gastric banding. Obes. Surg..

[55] Severinsen M.C.K., Pedersen B.K. (2020). Muscle-Organ Crosstalk: The Emerging Roles of Myokines. Endocr. Rev..

[56] Gomarasca M., Banfi G., Lombardi G. (2020). Myokines: The endocrine coupling of skeletal muscle and bone. Adv. Clin. Chem..

[57] Gonzalez-Gil A.M., Elizondo-Montemayor L. (2020). The Role of Exercise in the Interplay between Myokines, Hepatokines, Osteokines, Adipokines, and Modulation of Inflammation for Energy Substrate Redistribution and Fat Mass Loss: A Review. Nutrients.

[58] Yang M., Luo S., Yang J., Chen W., He L., Liu D., Zhao L., Wang X. (2022). Myokines: Novel therapeutic targets for diabetic nephropathy. Front. Endocrinol..

[59] Rebelos E., Dadson P., Oikonen V., Iida H., Hannukainen J.C., Iozzo P., Ferrannini E., Nuutila P. (2019). Renal hemodynamics and fatty acid uptake: Effects of obesity and weight loss. Am. J. Physiol. Endocrinol. Metab..

[60] Serra A., Esteve A., Navarro-Díaz M., López D., Bancu I., Romero R. (2015). Long-Term Normal Renal Function after Drastic Weight Reduction in Patients with Obesity-Related Glomerulopathy. Obes. Facts.

[61] Wang M., Wang Z., Chen Y., Dong Y. (2022). Kidney Damage Caused by Obesity and Its Feasible Treatment Drugs. Int. J. Mol. Sci..

[62] Xu T., Sheng Z., Yao L. (2017). Obesity-related glomerulopathy: Pathogenesis, pathologic, clinical characteristics and treatment. Front. Med..

[63] Mallamaci F., Ruggenenti P., Perna A., Leonardis D., Tripepi R., Tripepi G., Remuzzi G., Zoccali C. (2011). ACE inhibition is renoprotective among obese patients with proteinuria. J. Am. Soc. Nephrol..

[64] Valensi P., Derobert E., Genthon R., Riou J.P. (1996). Effect of ramipril on insulin sensitivity in obese patients. Time-course study of glucose infusion rate during euglycaemic hyperinsulinaemic clamp. Diabetes Metab..

[65] Che Y.Y., Hong H., Lei Y.T., Zou J., Yang Y.Y., He L.Y. (2022). ACE2 deficiency exacerbates obesity-related glomerulopathy through its role in regulating lipid metabolism. Cell Death Discov..

[66] Wei J., Ma C., Wang X. (2006). Simvastatin inhibits tissue factor and plasminogen activator inhibitor-1 expression of glomerular mesangial cells in hypercholesterolemic rabbits. Biomed. Res..

[67] Quiroga B., Muñoz Ramos P., Álvarez Chiva V. (2020). Efficacy and safety of the PCSK9 inhibitors in the treatment of dyslipidemia in chronic kidney disease. Nefrologia Engl. Ed..

[68] Zemheri-Navruz F., Ince S., Arslan-Acaroz D., Acaroz U., Demirel H.H., Demirkapi E.N. (2023). Resveratrol alleviates pyraclostrobin-induced lipid peroxidation, oxidative stress, and DNA damage in rats. Environ. Sci. Pollut. Res. Int..

[69] Gu W., Wang X., Zhao H., Geng J., Li X., Zheng K., Guan Y., Hou X., Wang C., Song G. (2022). Resveratrol ameliorates diabetic kidney injury by reducing lipotoxicity and modulates expression of components of the junctional adhesion molecule-like/sirtuin 1 lipid metabolism pathway. Eur. J. Pharmacol..

[70] Park H.S., Lim J.H., Kim M.Y., Kim Y., Hong Y.A., Choi S.R., Chung S., Kim H.W., Choi B.S., Kim Y.S. (2016). Resveratrol increases adipor1 and adipor2 expression in type 2 diabetic nephropathy. J. Transl. Med..

[71] Han Y., Xiong S., Zhao H., Yang S., Yang M., Zhu X., Jiang N., Xiong X., Gao P., Wei L. (2021). Lipophagy deficiency exacerbates ectopic lipid accumulation and tubular cells injury in diabetic nephropathy. Cell Death Dis..

[72] Gu W., Yang L., Wang X., Geng J., Li X., Zheng K., Guan Y., Hou X., Wang C., Song G. (2022). Pterostilbene, a Resveratrol Derivative, Improves Ectopic Lipid Deposition in the Kidneys of Mice Induced by a High-Fat Diet. Kidney Blood Press. Res..

[73] Barazzuol L., Giamogante F., Calì T. (2021). Mitochondria associated membranes (MAMs): Architecture and physiopathological role. Cell Calcium.

[74] Yang M., Li C., Yang S., Xiao Y., Xiong X., Chen W., Zhao H., Zhang Q., Han Y., Sun L. (2020). Mitochondria-Associated ER Membranes—The Origin Site of Autophagy. Front. Cell Dev. Biol..

[75] Yang M., Li C., Sun L. (2021). Mitochondria-Associated Membranes (MAMs): A Novel Therapeutic Target for Treating Metabolic Syndrome. Curr. Med. Chem..

[76] Yang M., Han Y., Luo S., Xiong X., Zhu X., Zhao H., Jiang N., Xiao Y., Wei L., Li C. (2021). MAMs Protect against Ectopic Fat Deposition and Lipid-Related Kidney Damage in DN Patients. Front. Endocrinol..

[77] Ni L., Yuan C., Chen G., Zhang C., Wu X. (2020). SGLT2i: Beyond the glucose-lowering effect. Cardiovasc. Diabetol..

[78] González-Albarrán O., Morales C., Pérez-Maraver M., Aparicio-Sánchez J.J., Simó R. (2022). Review of SGLT2i for the Treatment of Renal Complications: Experience in Patients with and without T2D. Diabetes Ther..

[79] Santos-Gallego C.G., Vargas-Delgado A.P., Requena-Ibanez J.A., Garcia-Ropero A., Mancini D., Pinney S., Macaluso F., Sartori S., Roque M., Sabatel-Perez F. (2021). Randomized Trial of Empagliflozin in Nondiabetic Patients with Heart Failure and Reduced Ejection Fraction. J. Am. Coll. Cardiol..

[80] Wheeler D.C., Toto R.D., Stefánsson B.V., Jongs N., Chertow G.M., Greene T., Hou F.F., Mcmurray J.J.V., Pecoits-Filho R., Correa-Rotter R. (2021). A pre-specified analysis of the DAPA-CKD trial demonstrates the effects of dapagliflozin on major adverse kidney events in patients with IgA nephropathy. Kidney Int..

[81] Rådholm K., Figtree G., Perkovic V., Solomon S.D., Mahaffey K.W., De Zeeuw D., Fulcher G., Barrett T.D., Shaw W., Desai M. (2018). Canagliflozin and Heart Failure in Type 2 Diabetes Mellitus: Results from the CANVAS Program. Circulation.

[82] Wu J., Sun Z., Yang S., Fu J., Fan Y., Wang N., Hu J., Ma L., Peng C., Wang Z. (2022). Kidney single-cell transcriptome profile reveals distinct response of proximal tubule cells to SGLT2i and ARB treatment in diabetic mice. Mol. Ther..

[83] Nelson A.J., Pagidipati N.J., Aroda V.R., Cavender M.A., Green J.B., Lopes R.D., Al-Khalidi H., Gaynor T., Kaltenbach L.A., Kirk J.K. (2021). Incorporating SGLT2i and GLP-1RA for Cardiovascular and Kidney Disease Risk Reduction: Call for Action to the Cardiology Community. Circulation.

[84] Kaze A.D., Zhuo M., Kim S.C., Patorno E., Paik J.M. (2022). Association of SGLT2 inhibitors with cardiovascular, kidney, and safety outcomes among patients with diabetic kidney disease: A meta-analysis. Cardiovasc. Diabetol..

[85] Xu L., Ota T. (2018). Emerging roles of SGLT2 inhibitors in obesity and insulin resistance: Focus on fat browning and macrophage polarization. Adipocyte.

[86] Martínez-Montoro J.I., Morales E., Cornejo-Pareja I., Tinahones F.J., Fernández-García J.C. (2022). Obesity-related glomerulopathy: Current approaches and future perspectives. Obes. Rev..

[87] Farkhondeh T., Llorens S., Pourbagher-Shahri A.M., Ashrafizadeh M., Talebi M., Shakibaei M., Samarghandian S. (2020). An Overview of the Role of Adipokines in Cardiometabolic Diseases. Molecules.

[88] Fève B., Bastard C., Fellahi S., Bastard J.P., Capeau J. (2016). New adipokines. Ann. Endocrinol..

[89] Zhang Y., Proenca R., Maffei M., Barone M., Leopold L., Friedman J.M. (1994). Positional cloning of the mouse obese gene and its human homologue. Nature.

[90] Obradovic M., Sudar-Milovanovic E., Soskic S., Essack M., Arya S., Stewart A.J., Gojobori T., Isenovic E.R. (2021). Leptin and Obesity: Role and Clinical Implication. Front. Endocrinol..

[91] Pereira S., Cline D.L., Glavas M.M., Covey S.D., Kieffer T.J. (2021). Tissue-Specific Effects of Leptin on Glucose and Lipid Metabolism. Endocr. Rev..

[92] Zhang F., Chen Y., Heiman M., Dimarchi R. (2005). Leptin: Structure, function and biology. Vitam. Horm..

[93] Suriano F., Vieira-Silva S., Falony G., Roumain M., Paquot A., Pelicaen R., Régnier M., Delzenne N.M., Raes J., Muccioli G.G. (2021). Novel insights into the genetically obese (ob/ob) and diabetic (db/db) mice: Two sides of the same coin. Microbiome.

[94] Niu K., Bai P., Yang B., Feng X., Qiu F. (2022). Asiatic acid alleviates metabolism disorders in ob/ob mice: Mechanistic insights. Food Funct..

[95] Straub L.G., Scherer P.E. (2019). Metabolic Messengers: Adiponectin. Nat. Metab..

[96] Ruiyang B., Panayi A., Ruifang W., Peng Z., Siqi F. (2021). Adiponectin in psoriasis and its comorbidities: A review. Lipids Health Dis..

[97] Xu X., Huang X., Zhang L., Huang X., Qin Z., Hua F. (2021). Adiponectin protects obesity-related glomerulopathy by inhibiting ROS/NF-Κb/NLRP3 inflammation pathway. BMC Nephrol..

[98] Yang M., Song P., Zhao L., Wang X. (2023). Adipose-Renal Axis in Diabetic Nephropathy. Curr. Med. Chem..

[99] Wang X.X., Edelstein M.H., Gafter U., Qiu L., Luo Y., Dobrinskikh E., Lucia S., Adorini L., D’Agati V.D., Levi J. (2016). G Protein-Coupled Bile Acid Receptor TGR5 Activation Inhibits Kidney Disease in Obesity and Diabetes. J. Am. Soc. Nephrol..

[100] Musso G., Cassader M., Cohney S., De Michieli F., Pinach S., Saba F., Gambino R. (2016). Fatty Liver and Chronic Kidney Disease: Novel Mechanistic Insights and Therapeutic Opportunities. Diabetes Care.

[101] Wilding J.P.H., Batterham R.L., Calanna S., Davies M., Van Gaal L.F., Lingvay I., Mcgowan B.M., Rosenstock J., Tran M.T.D., Wadden T.A. (2021). Once-Weekly Semaglutide in Adults with Overweight or Obesity. N. Engl. J. Med..

[102] Gerstein H.C., Colhoun H.M., Dagenais G.R., Diaz R., Lakshmanan M., Pais P., Probstfield J., Riesmeyer J.S., Riddle M.C., Rydén L. (2019). Dulaglutide and cardiovascular outcomes in type 2 diabetes (REWIND): A double-blind, randomised placebo-controlled trial. Lancet.

[103] Xu Y., Fu J.F., Chen J.H., Zhang Z.W., Zou Z.Q., Han L.Y., Hua Q.H., Zhao J.S., Zhang X.H., Shan Y.J. (2018). Sulforaphane ameliorates glucose intolerance in obese mice via the upregulation of the insulin signaling pathway. Food Funct..

[104] Huo L., Su Y., Xu G., Zhai L., Zhao J. (2019). Sulforaphane Protects the Male Reproductive System of Mice from Obesity-Induced Damage: Involvement of Oxidative Stress and Autophagy. Int. J. Environ. Res. Public. Health.

[105] Lu Y., Zhang Y., Lou Y., Cui W., Miao L. (2020). sulforaphane suppresses obesity-related glomerulopathy-induced damage by enhancing autophagy via Nrf2. Life Sci..

[106] Ren Y., Wang D., Lu F., Zou X., Xu L., Wang K., Huang W., Su H., Zhang C., Gao Y. (2018). Coptidis rhizoma inhibits nlrp3 inflammasome activation and alleviates renal damage in early obesity-related glomerulopathy. Phytomedicine.

[107] Guo Y.P., Jiang H.K., Jiang H., Tian H.Y., Li L. (2018). Lipoxin A4 may attenuate the progression of obesity-related glomerulopathy by inhibiting NF-Κb and ERK/P38 MAPK-dependent inflammation. Life Sci..

